# Effects of cycling on lithium-ion battery hysteresis and overvoltage

**DOI:** 10.1038/s41598-019-51474-5

**Published:** 2019-10-16

**Authors:** V. J. Ovejas, A. Cuadras

**Affiliations:** Grup de Processat d’Energia i Circuits Integrats (EPIC), Departament d’Enginyeria Electronica, Escola d’Enginyeria de Barcelona Est (EEBE), Universitat Politècnica de Catalunya - BarcelonaTech, Barcelona, Spain

**Keywords:** Energy, Energy storage, Batteries

## Abstract

Currently, lithium-ion batteries are widely used as energy storage systems for mobile applications. However, a better understanding of their nature is still required to improve battery management systems (BMS). Overvoltages and open-circuit voltage (OCV) hysteresis provide valuable information regarding battery performance, but estimations of these parameters are generally inaccurate, leading to errors in BMS. Studies on hysteresis are commonly avoided because the hysteresis depends on the state of charge and degradation level and requires time-consuming measurements. We have investigated hysteresis and overvoltages in Li(NiMnCo)O_2_/graphite and LiFePO_4_/graphite commercial cells. Here we report a direct relationship between an increase in OCV hysteresis and an increase in charge overvoltage when the cells are degraded by cycling. We find that the hysteresis is related to diffusion and increases with the formation of pure phases, being primarily related to the graphite electrode. These findings indicate that the graphite electrode is a determining factor for cell efficiency.

## Introduction

Currently, lithium-ion batteries are prevalent in power mobile applications because their energy- and power-to-weight ratios are higher than those of other energy storage systems. The primary drawbacks of lithium-ion batteries include the charging time and performance degradation, which compromise their lifetime. Degradation and internal losses worsens the performance of battery-monitoring systems (BMSs) with cycling^1^, where BMSs rely on the open-circuit voltage (OCV), operational voltage (*V*_cell_) and delivered charge. The OCV is the thermodynamic voltage provided by the battery chemistry, and the operational voltage is the voltage at the battery terminals. Losses in batteries are attributed to the overvoltage and OCV hysteresis observed during charging and discharging.

The overvoltage accounts for internal losses, which reflect electrical and ionic conduction through various components of the cell and charge transfer phenomena^2,3^. In some studies, side reactions, such as solid–electrolyte interface (SEI) formation, have also been considered as overvoltage^4^. Therefore, a change in overvoltage with battery ageing is expected because the reaction kinetics and transport characteristics are altered with ageing, as has been commonly reported in the literature^5–7^. In a previous study, we demonstrated that the increase in discharge overvoltage during cycling of NMC cells is attributed to an increase in diffusion overvoltage, with a direct correspondence between the overvoltage and phase transformations in the electrodes^8^. A relationship among the increase in overvoltage, diffusion and phase formations has been also reported in the literature^9,10^.

The electrodes in Li-ion batteries relax to different OCV values after lithiation and delithiation, even for the same stoichiometry. OCV hysteresis is defined as the difference between the lithiation and delithiation OCVs^11–13^. Hysteresis results from thermodynamic entropic effects, mechanical stress and microscopic distortions within the active material particles^13^. Thermodynamic hysteresis is associated with electrodes composed of numerous active material particles^12,13^ and is attributed to the varying lithium insertion rates for the particles within a comprehensive electrode^13^. Mechanical hysteresis can occur simultaneously with thermodynamic hysteresis^11,12^. Mechanical hysteresis is caused by the different lattice constants of lithiated and delithiated phases, which cause mechanical stress at the phase barrier^13^. Mechanical effects are related to volume changes, surface tension and other mechanical effects. In particular, mechanical hysteresis is associated with reversible^14–16^ and irreversible^17–19^ processes and a combination of both^14–16^. In some studies, a decrease in hysteresis is observed with an increase in the applied current^13^. One possible mechanism of this behaviour is the interparticle charge transfer caused by strong inhomogeneities in lithium content between particles at high rates^13^.

Despite the available research related to hysteresis, controversy remains regarding the causes and effects of hysteresis. When structural defects, such as distortion, plastic deformation or cracks, occur, changes in the electrical, thermal and mechanical properties and in the ionic diffusion and conduction of the electrodes are expected. Currently, studies on the effect of hysteresis are commonly avoided in commercial Li-ion applications because hysteresis measurements are time-consuming, with the hysteresis depending on the battery degradation and state of charge (SoC). If the increase in hysteresis could be determined from the increase in overvoltage, which can be obtained from data that are already available in the BMS, the time required for updating the OCV hysteresis in practical applications would be drastically reduced. In this paper, we aim to find a relationship between the variation in overvoltage with ageing, which reflects the transport and transfer characteristics of the cells, and OCV hysteresis.

## Results

### Overvoltage and OCV hysteresis in NMC cells

**Figure 1:**
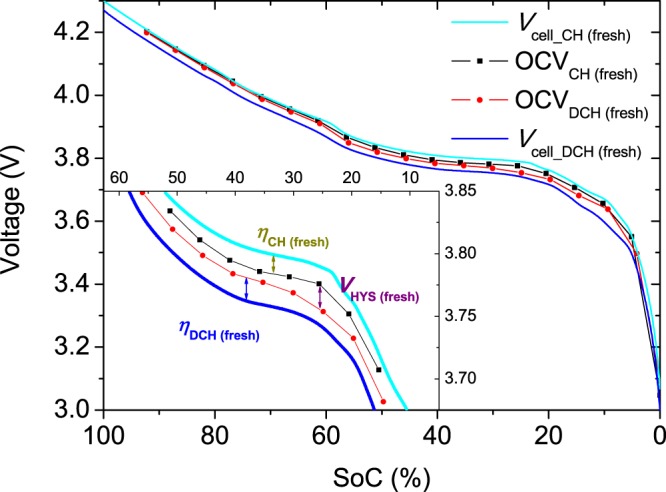
Charge and discharge cell voltages of NMC cells at a rate of C/25 and OCVs corresponding to a fresh NMC cell. The inset shows how the overvoltages and voltage hysteresis are obtained.

**Figure 2:**
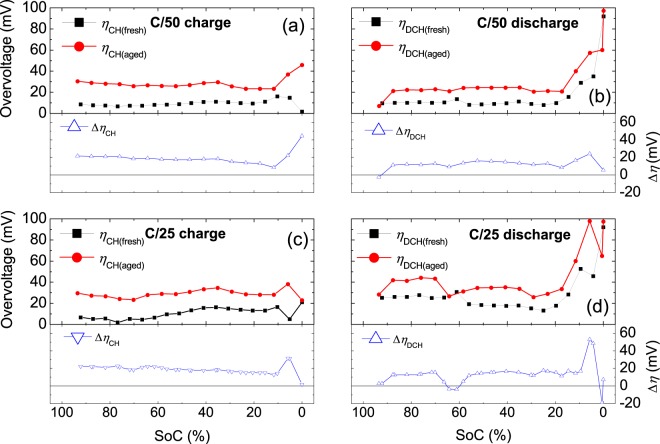
Overvoltages generated in NMC cells at C/50 during (**a**) charging and (**b**) discharging and at C/25 during (**c**) charging and (**d**) discharging. The increases in overvoltage after a cycle-ageing test are shown in the bottom panels.

The overvoltages generated in NMC cells during charging (*η*_CH_) and discharging (*η*_DCH_) and the OCV hysteresis (*V*_HYS_) are obtained from the difference between the cell voltages and OCVs (Fig. 1), as described in the Methods section. The overvoltages are calculated at low rates (C/50 and C/25) for fresh and aged cells (Fig. 2). In general, we find that the generated overvoltage increases during cycling, except for particular SoC values, such as those near 60%, when discharging at C/25 (Fig. 2d).

We find that the overvoltage depends on the charge and discharge rates, as expected from the literature^20^. However, we note that the overvoltage rate dependence is also SoC dependent. For instance, this dependence can be clearly observed in the discharge overvoltage of an aged cell if we compare the results for an SoC of 64% at C/50 (Fig. 2b) and C/25 (Fig. 2d), in which a large variation is observed compared with neighbouring SoCs.

**Figure 3:**
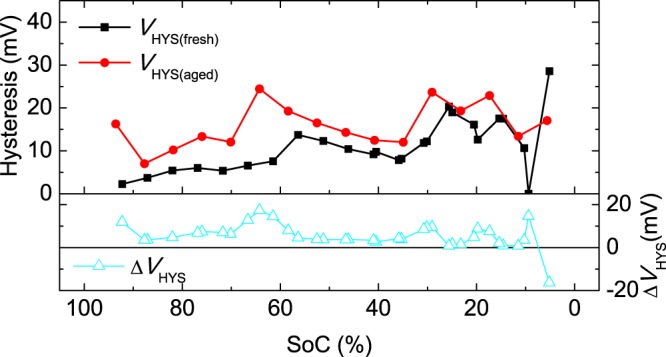
Hysteresis observed in fresh and aged NMC cells and the corresponding increase after the ageing test.

We find that the OCV hysteresis is larger for the aged cells (Fig. 3), except for SoCs, of approximately 25% and 13%, for which there is no variation with ageing. For both ageing states, various peaks are observed in the curves, which are related to a larger hysteresis at some particular SoCs. We find that these peaks tend to shift to higher SoCs as the cell ages.

**Figure 4:**
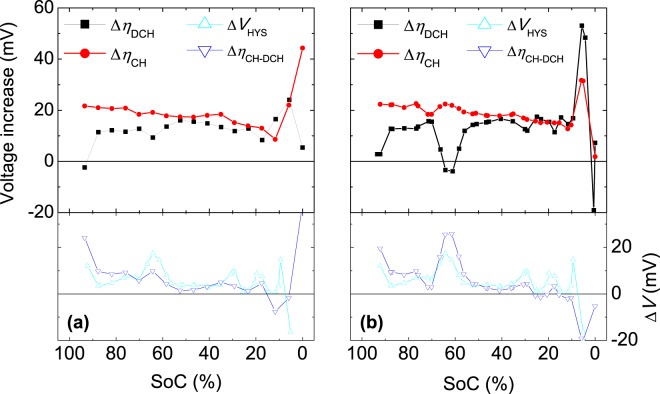
Increase in overvoltage and hysteresis after degradation of the NMC cell by cycling, evaluated at (**a**) C/50 and (**b**) C/25.

In general, the increase in overvoltage is larger during charging than during discharging (Fig. 4), indicating that more energy is lost during charging as the cells age, which reduces their energy efficiency. Interestingly, we find that the larger increase in overvoltage during charging compared with discharging (determined from the difference between the two contributions) shows a direct correspondence with the increase in hysteresis (blue and green open symbols in Fig. 4, respectively). Moreover, some differences are observed between the results for C/25 and C/50 (Fig. 4a,b). The most evident difference is detected at SoCs near 64%, at which the increase in charge overvoltage obtained at the higher rate of C/25 (Fig. 4b) is larger than that obtained at the lower rate of C/50 (Fig. 4a). This difference may be attributed to a kinetic artefact introduced by the larger current for C/25. Smaller differences between the curves (on the order of a few millivolts) are observed outside this SoC range.

### Overvoltage and OCV hysteresis in LFP cells

**Figure 5:**
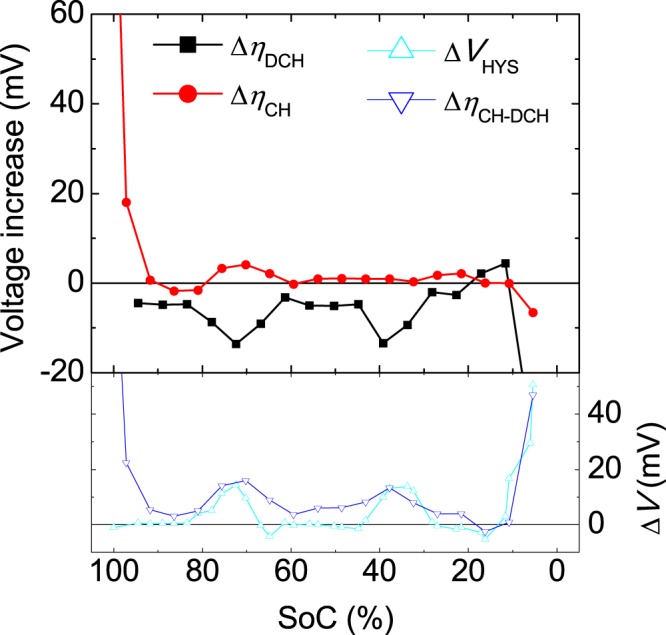
Increase in overvoltage and hysteresis after degradation of the LFP cell by cycling, evaluated at C/25.

The same analysis was conducted in an LFP/graphite cell in order to generalise the results obtained for the NMC/graphite cells. The results for the LFP cells show that the charge overvoltage increases with ageing, whereas the discharge overvoltage decreases (Fig. 5). In a previous study, we demonstrated that the discharge overvoltage decreases during the first 300 cycles (due to a decrease in impedance) and then increases until the end of the ageing test (cycle 1049)^21^. However, the increase experienced by the cells during the cycling test is not sufficient to exceed the initial discharge overvoltage. Thus, if intermediate cycles are not evaluated, one might wrongly conclude that the discharge overvoltage decreases with cycling, whereas in reality, the overvoltage has been increasing for more than 700 cycles. For this particular chemistry, initial decreases in overvoltage or impedance have been commonly reported in the literature^22–24^. The decreased impedance and increased capacity at high rates upon initial stages of cycling have been previously attributed to electrochemical milling or decrepitation of the LFP particles^25,26^. However, the aim of this paper is not to determine the origin of this decrease but whether there is any relation between overvoltage and hysteresis.

If we compare the hysteresis increase during the cycling test (Δ*V*_HYS_) with the difference between the increases in charge and discharge overvoltages (Δ*η*_CH-DCH_), we find a correspondence between the two parameters (Fig. 5), as observed for the NMC cells (Fig. 4). Although the increase in discharge overvoltage is negative, the larger increase in charge overvoltage follows the same tendency as the hysteresis increase, and both terms have similar values. Larger differences can be observed at SoC values above 95%.

### OCV hysteresis and phase transformations in NMC cells

**Figure 6:**
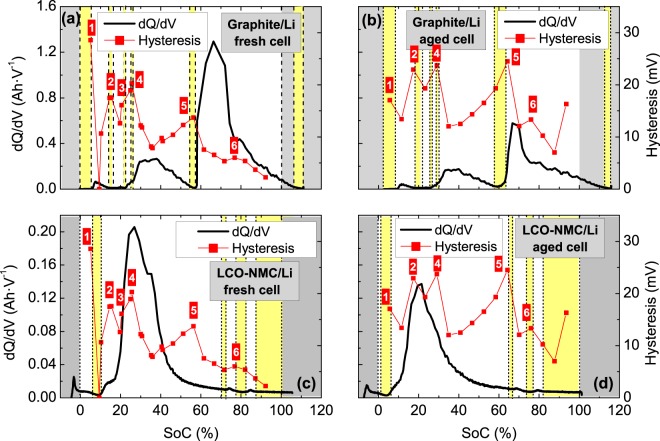
Incremental capacity of the (**a**,**b**) graphite and (**c**,**d**) NMC electrodes configured as half-cells during charging at C/25 and OCV hysteresis at room temperature. (**a**,**c**) represent fresh electrodes, and (**b**,**d**) represent aged electrodes. The yellow areas represent single-phase regions, and the grey areas delineate the operating SoC of the full cell.

The SoCs corresponding to the formation of various phases through which the electrodes pass during the charging process were determined by an incremental capacity analysis (highlighted in yellow and separated by vertical dashed lines in Fig. 6). A correspondence is found between the SoCs at which hysteresis tends to increase and the SoCs at which the phases are formed (Fig. 6). In particular, six peaks (or discontinuities) are detected in the hysteresis for the fresh cell (numerically labelled in Fig. 6a,c), whereas only five peaks can be identified for the aged cell (Fig. 6b,d). The peaks labelled as 2, 3 and 4 coincide with the formation of graphite stages (Fig. 6a). In a previous work, we attributed the phase changes coinciding with peaks 2, 3, 4 and 5 to the negative electrode^8^. In particular, these changes were associated with the formation of 4L, 3L, 2L and 2 stages of graphite^8^. Nevertheless, we find that peak 6 coincides with a phase transformation in the lithium oxide electrode (Fig. 6c). Unfortunately, no information can be provided about the particular phase to which the electrode transforms at these SoCs. Unlike the other peaks, the origin of peak 1 is not clear because it coincides with the formation of phases at both electrodes (Fig. 6a,c), as will be discussed in detail in the following section.

### Similarities in OCV hysteresis in NMC and LFP cells

**Figure 7:**
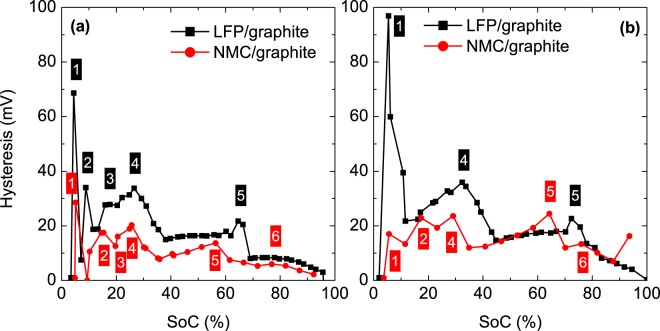
OCV hysteresis in (**a**) fresh and (**b**) aged NMC and LFP cells. The identified peaks are numerically labelled from the lowest to highest SoC.

Similarities in hysteresis are expected between the two cells considered in this study (NMC and LFP), as both cells contain graphite as a negative electrode. Therefore, the hysteresis measured for the NMC cells was compared with that for the LFP cells (Fig. 7). The same pattern was observed (Fig. 7a), with an exact match for the peak positions and amplitudes, except for the peak at the lowest SoC (peak 1), was reported by Barai *et al*. for a cell composed of LFP and graphite^27^. We found that peaks 1–5 appear in both evaluated cells (NMC and LFP), particularly in the fresh cells (Fig. 7a). These results indicate that peaks 1–5 correspond to the graphite electrode. Graphite passes through five different phases during lithiation and delithiation, which correspond to the five peaks identified in our results (Fig. 7a). For the NMC cells, the only peak that could not be associated with a particular electrode in Fig. 6 was peak 1 because single phases were formed in both electrodes. From the comparison with the LFP cell, it is clear that peak 1 corresponds to the graphitic electrode (Fig. 7). Despite a clear identification of the peaks corresponding to graphite, the peaks occur at different SoCs and exhibit different amplitudes depending on the chemistry.

## Discussion

We found that the increase in overvoltage at very low rates (C/50 and C/25) after the degradation process (Δ*η*_CH_ and Δ*η*_DCH_) coincide for both charging and discharging, but an additional term arises during charging. This additional term is directly related to the hysteresis. This affirmation is valid for both chemistries, NMC and LFP, as inferred from Figs 4 and 5. As a physical interpretation, it is assumed that one component of the transport and charge transfer is equally affected by ageing during charging and discharging, whereas another component of the transport and charge transfer (and possible side reactions) is affected only during charging. In general, the increase in overvoltage with cycling can be explained as the transport of ions becoming more inefficient (i.e. dissipating more energy) and/or the charge transfer slowing, which also dissipates more energy. This effect arises from the consumption of additional energy during the charging process, as the cell is cycled. Consequently, the final state of the cell differs depending on the direction of the current, as reflected by the hysteresis. We conclude that the additional energy required for recharging the battery after cycling is due to the increase in hysteresis, as can be inferred from the results (Figs 4 and 5).

The assumption of side reactions may be appropriate because the voltage during charging is larger than that during discharging, resulting in a greater possibility for overcoming the activation energy of secondary reactions. However, the degradation of the transport and transfer characteristics may be produced by changes in the conductive properties of any of the elements comprising the cell or by the reduced rate of the electrochemical reaction. For the SoCs at which the single phases are formed, there is a sharp increase in overvoltage^8^ and hysteresis (Fig. 6). The increase in overvoltage towards the end of a phase transformation has been associated with ionic transport, particularly diffusion^8^. Thus, the results indicate that hysteresis and diffusion are closely related. Possible causes of the deterioration of the transport properties of the cells include wearing of the components, loss of contact or defect formation in the electrode structure. In particular, defects in the electrodes have been related to hysteresis^9,19,28^. Irreversible processes associated with hysteresis include mechanical strain^9,29^, particle fractures^29^, the formation of dislocations^9,19,28^, structural disordering^28^, boundary motion and plastic deformation^19,28^ and volume changes, dislocations or the presence of impurities^19^. Some factors that contribute to hysteresis and enhance the morphological changes include electrochemical inhomogeneities in phase and composition^29^, the movement of an interface^19^ or the strain accommodation energy of phase transformations induced from the volume changes between lithiated and delithiated phases^9,19^. Therefore, the occurrence of morphological changes due to cycling may be responsible for the coincidence in overvoltage and hysteresis increases. In fact, electrode defects produced during cycling (primarily during the charging process) have been reported in the literature^10,29,30^.

Hysteresis is generally not considered in practical applications because it usually accounts for a small portion of the total loss. However, the neglect of hysteresis is a source of error, which accumulates during battery operation. As we have shown in this paper, valuable information can be obtained from the hysteretic behaviour of a system in terms of energy efficiency and mechanical degradation. For greater accuracy in practical applications, we aim to facilitate an online calculation of the OCV hysteresis by defining a relationship between hysteresis and battery performance (from overvoltage measurements). Our results exhibit a direct relationship between the observed hysteresis increase with cycling and the overvoltage increase with ageing. Furthermore, the same behaviour has been observed in two different chemistries, indicating a possible generalisation of this technique. This generalisation would enable updating of the OCV hysteresis as cells are cycled in real applications.

## Methods

### Evaluated cells

We analysed two types of commercial Li-ion cells with LiCoO_2_–Li(NiMnCo)O_2_ (LCO-NMC or simply NMC) and LiFePO_4_ (LFP) as the positive electrode and graphite as the negative electrode. The NMC cells had a capacity of 2.8 Ah and were supplied by LG_Chem in the 18650 format. The expected cycle life reported by the manufacturer at which the cell maintains a capacity equal to or higher than 78% of the nominal capacity is 300 cycles when charging and discharging at C/2 at a temperature of 23 °C ± 2 °C.

LFP cells were provided by AA Portable Power Corp in the 14430 format with a nominal capacity of 0.4 Ah. The expected cycle life reported by the manufacturer at which the cell maintains a capacity equal to or higher than 70% of the nominal capacity is 1000 cycles when charging and discharging at a symmetric rate of C/5 at a temperature of 20 °C ± 5 °C.

### Cycling

To evaluate the cells at various ageing levels, the cells were subjected to a cycle-ageing process. The cells were charged at a constant current and voltage at the recommended C-rate and discharged at the maximum discharge C-rate allowed by the manufacturer.

NMC cells were charged at C/2 and discharged at 3C/2 in an HVBT 5560 Arbin Tester capable of measuring current and voltage. The cells were subjected to 350 cycles in a climatic chamber at 25 °C. The capacity fade experienced by the cells during discharging at C/25 and 3 C/2 was 15% and 20%, respectively.

LFP cells were charged at C/5 and discharged at 2C. The cells were cycled 1000 times in a climatic chamber at 25 °C. The LFP cells were cycled in two steps with two different instruments. The first cycling step was performed in an HVBT 5560 Arbin Tester, and the second cycling step was conducted with a VSP Bio-logic potentiostat/galvanostat under the same conditions. The temperature was controlled with a laboratory-cooled incubator, ILW53, provided by Pol-Eko Aparatura. The capacity fade experienced by the cells during discharging at C/25 and 2C was 23% and 15%, respectively.

Prior to the cycle-ageing test, the cells underwent six formation cycles at 25 °C to stabilise their capacity and to allow the initial formation of the SEI layer. Thus, we consider the cells as fresh immediately after the formation cycles (fresh cells) and as aged at the end of the cycle-ageing procedure (aged cells). The capacity fade experienced by the NMC cells at a discharging rate of 3 C/2 was 24% with respect to the nominal capacity and 15% in the case of the LFP cells, evaluated at a discharge rate of 2 C. A more detailed description of the ageing process can be found elsewhere^21^.

### OCV and hysteresis

The OCV measurements were based on the galvanostatic intermittent titration technique (GITT), which is commonly used to measure the OCV of batteries^9,31^. The GITT consists of charging and discharging the cell with pulses followed by relaxation periods. At the end of each relaxation period, the OCV is measured. GITT measurements were carried out during charging and discharging, resulting in two OCV curves: one for charging (OCV_CH_) and one for discharging (OCV_DCH_). The difference between the charging and discharging OCV curves is termed the OCV hysteresis (*V*_HYS_)^27,32,33^:

1 $$OC{V}_{CH}-OC{V}_{DCH}={V}_{HYS}$$

In this study, the GITT was performed with 150-min charge/discharge pulses at C/50 followed by a 16-h resting period. To maintain a constant starting SoC, four charge/discharge cycles at C/50 with a constant current were applied, followed by a remnant capacity procedure at C/50 prior to the GITT analysis. Measurements were acquired with a VSP potentiostat/galvanostat provided by Bio-logic. During the GITT tests, the cells were configured for two electrode measurements at four points, in which voltage measurements were carried out closer to the electrodes than the current injection. The cells were maintained at 25 °C in an IL53 incubator provided by Pol-Eko Aparatura.

### Overvoltage calculation

The overvoltage was calculated as the difference between the OCV obtained by the GITT technique and the cell voltage under polarisation (*V*_cell_) during discharging (*η*_DCH_) and charging (*η*_CH_):

2 $${\eta }_{DCH}=OC{V}_{DCH}-{V}_{cell\_DCH}$$

3 $${\eta }_{CH}={V}_{cell\_CH}-OC{V}_{CH}$$

### OCV hysteresis and overvoltage increases

The increases in hysteresis and overvoltage due to cycling were obtained as the difference between the aged cell values and the fresh cell values. For the hysteresis, the increase was obtained for each SoC value (Δ*V*_HYS_), as the difference between the hysteresis measured for the aged cell (*V*_HYS(aged)_) and that for the fresh cell (*V*_HYS(fresh)_):

4 $$\Delta {V}_{{\rm{HYS}}}={V}_{{\rm{HYS}}({\rm{aged}})}-{V}_{{\rm{HYS}}({\rm{fresh}})}$$

The increase in overvoltage was obtained for each SoC value during discharging and charging (Δ*η*_DCH_ and Δ*η*_CH_) as the difference between the overvoltage produced in the aged cell (*η*_DCH(aged)_ and *η*_CH(aged)_, respectively) and that in the fresh cell (*η*_DCH(fresh)_ and *η*_CH(fresh)_):

5 $$\Delta {\eta }_{{\rm{DCH}}}={\eta }_{{\rm{DCH}}({\rm{aged}})}-{\eta }_{{\rm{DCH}}({\rm{fresh}})}$$

6 $$\Delta {\eta }_{{\rm{CH}}}={\eta }_{{\rm{CH}}({\rm{aged}})}-{\eta }_{{\rm{CH}}({\rm{fresh}})}$$

In general, we found that the overvoltage increase was larger during charging. Thus, to represent the larger increase in overvoltage experienced by the cells during charging (Δ*η*_CH-DCH_), we calculated the difference between the increase during charging (Δ*η*_CH_) and the increase during discharging (Δ*η*_DCH_):

7 $$\Delta {\eta }_{{\rm{CH}}-{\rm{DCH}}}=\Delta {\eta }_{{\rm{CH}}}-\Delta {\eta }_{{\rm{DCH}}}$$

### Half-cells fabrication

Cell opening was applied to the NMC cells, as described elsewhere^21^. Cell opening was performed for one fresh cell after the formation cycles (fresh cell) and one aged cell after the cycle-ageing process (aged cell). Round samples with a diameter of 16 mm were harvested from positive and negative electrodes of the fresh and aged cells.

To build half-cells, one side of the active material of the harvested samples was removed by applying N-methyl-2-pyrrolidone (NMP) solvent by hand until the active material detached from the current collector. In particular, half-cells were built in a coin cell format by introducing the harvested positive or negative electrode, fresh separator, 90 μL of fresh electrolyte and metallic lithium as the counter electrode.

### Incremental capacity analysis

Incremental capacity analysis was performed for the half-cells at 25 °C during a charging process at C/25. Thus, the injected charge was measured as a function of the measured voltage. At each point, the incremental capacity was obtained as the portion of capacity associated with each voltage step (dQ/dV) and was represented as a function of the SoC.

Peaks in the incremental capacity curves indicate the coexistence of distinct phases (represented as plateaus in thermodynamic voltage curves). Each chemistry has a characteristic peak pattern, and each peak in the incremental capacity curve has a unique shape and intensity. In contrast, changes in slope in the thermodynamic voltage curve (transitions between plateaus) are related to the formation of pure or single phases^34–37^.

## Conclusions

We found that the hysteresis increases towards the end of phase transformations (i.e. the formation of pure phases) as well as the overvoltage, which has been previously associated with diffusion (transport characteristics). After ageing the cells by cycling, we found that the hysteresis increased and that the overvoltage exhibited a larger increase during charging than discharging. The most important finding of this work is a direct correspondence between the additional increase in overvoltage during charging and the increase in hysteresis after the cycle-ageing test. The same trend was detected in both NMC and LFP cells. We found that the increase in hysteresis due to cycle ageing is primarily related to the graphite electrode. In particular, mechanical degradation (i.e. structural modification of the cells) is a possible cause, as it would alter the transport characteristics (diffusion overvoltage) and would also be reflected in the OCV hysteresis.

## Data Availability

All data generated or analysed during this study are included in this published article.
